# Relationship between periodontitis and chronic obstructive pulmonary disease: a bibliometric analysis from 1945 to 2023

**DOI:** 10.4317/medoral.26582

**Published:** 2024-11-25

**Authors:** Ziyi Guo, Zuomin Wang

**Affiliations:** 1 MD, Department of Stomatology, Beijing Chaoyang Hospital, Capital Medical University, Beijing, China; 2 PhD, Department of Stomatology, Beijing Chaoyang Hospital, Capital Medical University, Beijing, China

## Abstract

**Background:**

Chronic obstructive pulmonary disease (COPD) and periodontitis are common chronic diseases. The presence of either of the two diseases increases the risk of the other, whereas managing one reduces the risk of the other. This study aimed to summarize the current state of research and trends in this field using bibliometric analysis and visualization.

**Material and Methods:**

We used PubMed to search and download all periodontal disease- and COPD-related studies published until August 20, 2023. We further performed bibliometric analysis on the text R and Python software and visualized the results using Gephi and VOSviewer to construct latent Dirichl*et al*location models that summarize idiosyncratic research themes.

**Results:**

A total of 2, 109 publications were analyzed, with recent ones focusing on risk factors and pandemics. The country that produced the most publications was the United States with 427 publications. The most cited article was by Prof. Wang Zuomin. International Journal of Chronic Obstructive Pulmonary Disease ranked first in publications. Keywords were focused on Risk Factors and Pandemics. In addition, COVID- 19, SARS-CoV-2 and coronavirus infections have become a research hotspot since 2020. However, little attention has been paid to environmental contamination and biological mechanisms.

**Conclusions:**

Research on periodontitis and COPD is expanding, and it currently focused on exploring risk factors and conducting clinical epidemiological studies. This exhaustive study provides a comprehensive summary of trends in this field and has important clinical implications for the screening and treatment of patients with COPD and periodontitis.

** Key words:**Chronic obstructive pulmonary disease, bibliometric analysis, inflammation, periodontitis, risk factors.

## Introduction

Chronic obstructive pulmonary disease (COPD) is a heterogeneous chronic disease manifesting as progressive restriction of airflow (1). Periodontitis, among the six most widespread noncommunicable diseases globally, impacts more than 40% of the world's population, with a prevalence rate of 54.8% in China (2,3). The relationship between these two diseases has recently become a research interest, because previous studies have shown that they may be closely related. Recent studies increasingly confirm the role of periodontitis in affecting overall health, potentially serving as a risk factor or indicator for COPD (4). Periodontitis and COPD share similar risk factors, such as smoking, microbial infections, environmental pollution, and poor oral hygiene practices (5). These factors may intertwine, leading to the occurrence and progression of both diseases. The pathophysiological processes of these two diseases are complex, and chronic inflammation and immune dysregulation play key roles in their occurrence and progression (6-8). Although several mechanisms have been proposed, the exact mechanism by which periodontitis is directly related to COPD remains to be elucidated.

Understanding the findings of previous periodontitis- and COPD-related studies can provide directions for future research. Bibliometrics can be used to provide visual representations of the impact of current research. It provides opportunities for objective data analysis that is more scalable and efficient than literature and systematic reviews, reducing the risk of bias that influences subjective decision-making. With the help of bibliometrics, it is possible to analyze the latest research trends and hot topics within a specific field of study. It is important for researchers to keep up with the latest scientific developments and plan their research accordingly. To the best of our knowledge, this is the first bibliometric analysis of the association between periodontitis and COPD. Our study aimed to analyze the overall characteristics, research hotspots, and future research trends in the association between periodontitis and COPD. A combination of bibliometrics allows for a more systematic understanding of the developmental landscape of this academic field as well as cutting-edge hotspots, creating future research opportunities for scientists and funding organizations and increasing awareness of current research bias.

## Material and Methods

- Data collection

We searched for all studies related to periodontal disease and COPD published on PubMed from its establishment to August 20, 2023. The following search terms were employed to retrieve literature from PubMed regardless of language or document type: Topic = (“periodontitis” OR “periodontal disease” OR “chronic periodontitis” OR “periodontal” OR “periodontics” OR “periodontology” OR “chronic generalized periodontitis” OR “ disease periodontal” OR “aggressive periodontitis ” OR “severe periodontitis” OR “progressive periodontitis” OR “generalized aggressive periodontitis” OR “advanced periodontitis” OR “localised periodontitis” OR “ ”) AND (“chronic obstructive pulmonary disease” OR “COPD” OR “chronic obstructive lung disease” OR “chronic obstructive respiratory disease ” OR “pulmonary disease，chronic obstructive) The search results were downloaded in XML format and preliminarily parsed using R software. Article titles, abstracts, keywords, and MeSH subject terms were extracted from the retrieved content. We conducted an additional investigation using the Web of Science database to gather comprehensive data on citations and top authors (9,10). The inclusion of Web of Science allowed us to capture a broader range of bibliometric indicators, such as citation counts and influential authors, which are critical for a detailed bibliometric analysis. This approach aligns with standard practices in bibliometric research, ensuring a more robust and thorough examination of the literature. By integrating data from both PubMed/MEDLINE and Web of Science, we aimed to enhance the accuracy and depth of our analysis, providing a more holistic view of the research landscape. This study adhered to the BIBLIO checklist guidelines, providing a structured framework for reporting bibliometric analyses (Supplement 1).

- Data analysis

The extracted text was preprocessed by performing word splitting, removing punctuation, and redundant words and using the term-frequency inverse document frequency (TF-IDF) method to calculate the word frequencies. We built a dictionary for constructing an implicit latent Dirichl*et al*location (LDA) model. LDA is a classical topic modeling method that extracts topics from a large amount of unstructured text and creates a vocabulary of features based on the frequency of the terms appearing in the text. The aim was to identify the specific research themes in each article.

We obtained a list of 30 themes, and for each article, we analyzed the abstract to identify the theme with the highest calculated probability. Clustering analysis was performed using Louvain's algorithm to construct a network of themes, from which relationships between themes were found, and clusters of related themes were created. Owing to its attribution to the two topics with the highest probability, the frequency of each topic in each article was calculated, and each topic was linked.

**Figure 1:**
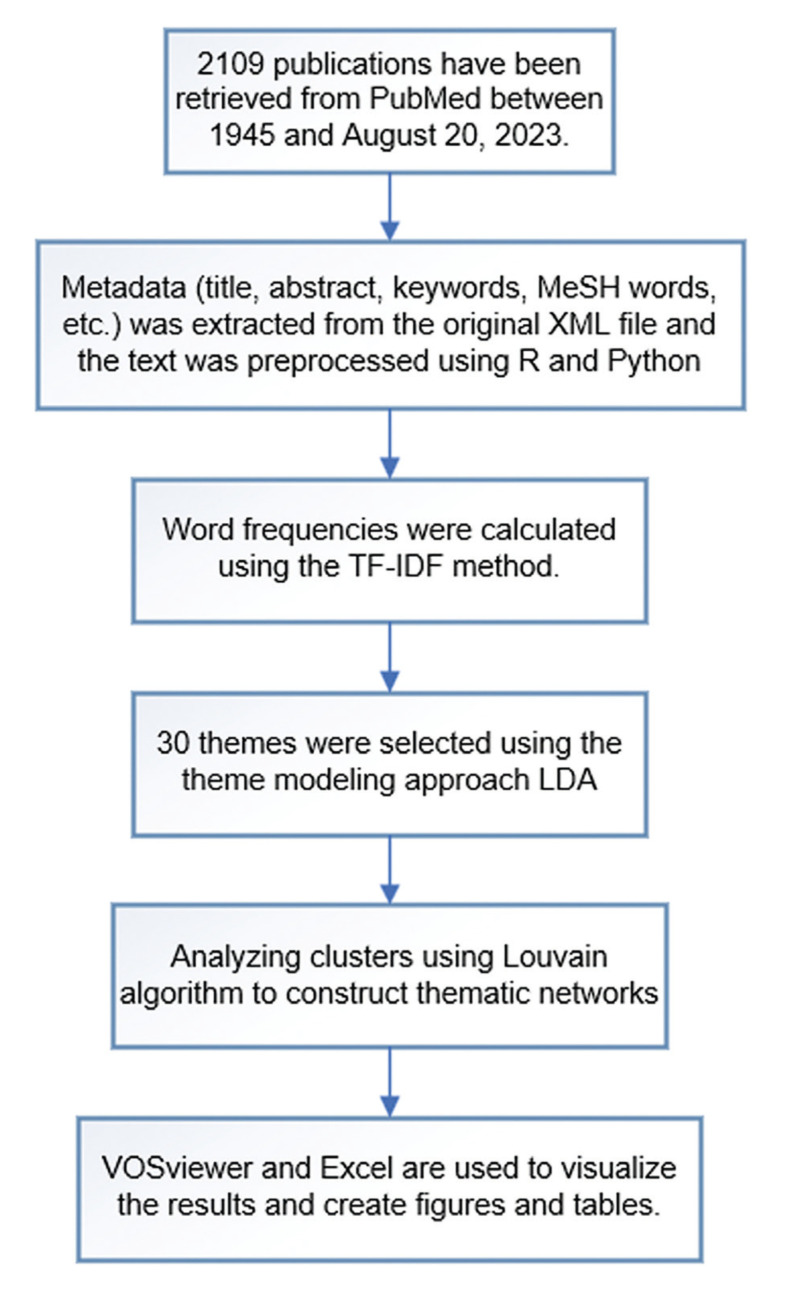
The flowchart illustrates the research process and strategy.

We analyzed the text using R 4.2. 1 and Python 3.7.0, constructed LDA models, mapped the topic networks using Gephi (https://gephi.org/), and VOSviewer software and Excel were utilized to visualize the results and draw the rest of the charts. Fig. 1 shows the flowchart.

## Results

- Analysis of authors/journals/countries/most cited publications

Among the highly productive authors, our research team stands out with the highest number of publications, totaling 10 papers from 1945 to August 2023. These publications have garnered 386 citations, averaging 38.6 citations per paper. Following closely is Song Yiqing, with 8 publications and 379 citations, averaging 47.38 citations per paper. As shown in Table 1, both researchers have collaborated on numerous occasions.

Their contributions to the field are significant, beginning with clinical research on the association between periodontitis and COPD. This work involved extensive investigations into the relationship between these diseases and their impacts, followed by *in vivo* and *in vitro* studies (11-14).

Supplement 2 presents the top 50 journals in both oral and respiratory fields based on the number of articles published. Notably, the International Journal of Chronic Obstructive Pulmonary Disease (Impact Factor [IF]: 2.8) and Chest (IF: 10.1) stand out, each with over 50 articles. These journals fall under the respiratory system category in the Journal Citation Reports, with 76 and 61 articles, respectively. The American Journal of Respiratory and Critical Care Medicine (IF: 24.7), recognized as a premier respiratory medicine journal, boasts the highest average citation count of 88.27 among the top journals, indicating its consistently high-quality publications. It's noteworthy that 5 of the top 10 journals are classified in Q1, while 2 are in Q2, 1 is in Q3 and 1 is in Q4 according to JCR.

The top 100 most cited publications in this study comprise 22 reviews, 6 meta-analyses, and 47 articles (Table 2). Among them, the top three citations include 2 reviews and 1 article, authored by Jeppe Madura Larsen, Amir Azarpazhoo and Frank A. Scannapieco, with citation frequencies of 537, 375, and 354, respectively. These findings underscore the authors' significant contributions to the field, as their studies are widely referenced by researchers. Notably, our research group has contributed three clinical research articles on periodontitis and COPD, aligning with the analysis of the authors.

**Figure 2:**
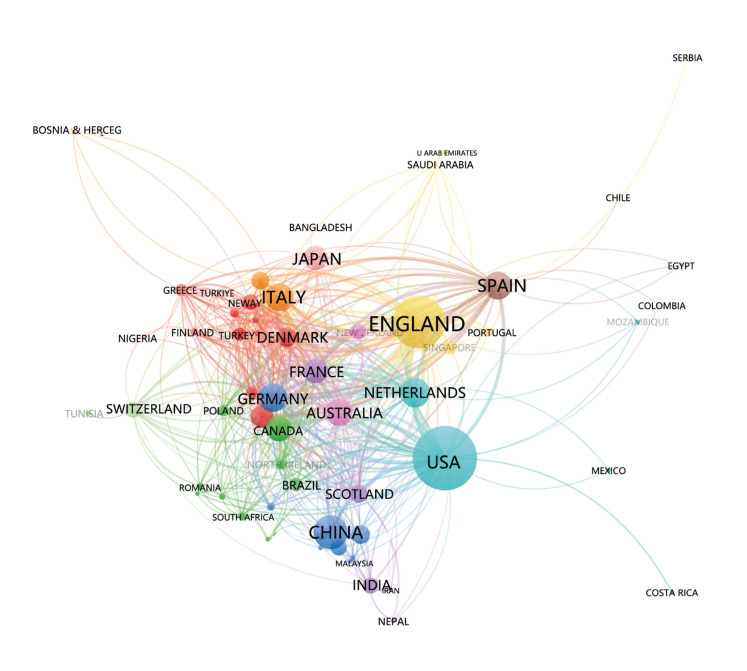
VOSviewer network visualization map of countries.

Furthermore, our analysis encompassed 62 countries based on their publication output in periodontitis and COPD research. Initially, countries with a minimum of 2 articles were visualized and assessed using VOSviewer, as depicted in Fig. 2. In the visualization, larger nodes represent higher article output, while the thickness of connecting lines signifies the strength of collaboration between countries. Node color indicates distinct clusters. Remarkably, the United States, England, and China emerge as pivotal players in the global research network, engaging in extensive collaboration with numerous countries.

Supplement 3 provides a detailed analysis of the top 5 countries contributing to research in this field. American researchers lead the field with 427 publications, amassing a substantial citation count of 21,266. Following closely, England boasts 302 publications and a commendable citation count of 16,858, surpassing even the United States in this regard. China ranks third with 140 articles and 3,055 citations.

- MeSH analysis

In this study, all MeSH words in the literature were analyzed. First, MeSH words that specifically referred to disease names (e.g., “Periodontal Diseases”) were excluded based on previous literature. The remaining MeSH words were classified into two categories: 1) MeSH words that specifically referred to the study subject (e.g., “Human”), and 2) MeSH words other than those that specifically referred to the study subject.

Seven age groups were defined in this study based on MeSH words: infants (aged 2- 3 months), preschoolers (aged 2-5 years), children (aged 6- 12 years), adolescents (aged 13- 18 years), adults (aged 19-44 years), middle-aged adults (aged 45-64 years), and older adults (aged ≥65 years). Articles involving research participants from any age group were included in each group. Overall, literature involving adults and middle-aged adults constituted the largest percentage of this study content, followed by literature related to older adults. Table 3 lists the top 20 occurrences of MeSH words in the literature. “COVID- 19,” “SARS-CoV-2,” and “Pandemics” associated with new crowns were the top three, followed by “Risk Factors” and “Animals.” Table 4 lists the top 15 MeSH words used every 10 years.

- Theme analysis

**Figure 3:**
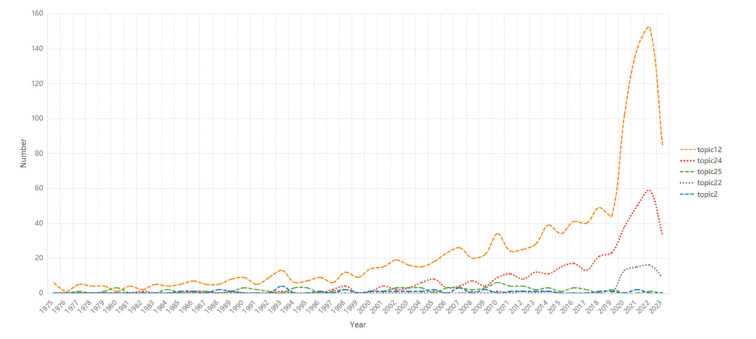
LDA analysis: annual trends in the top five popular topics. Topic 12: "treatment”, "patients”, and “infection”; topic 24: “treatment,” “patients”, and “infection”; topic 2: “health”, “risk”, and “smoking”; topic 25: “metastatic” and "lesions”; topic 22: “covid”, “pandemic”, and “studies". Number in the Y axis indicated the number of published studies. LDA, latent Dirichl*et al*location.

Based on literature summaries, the LDA method extracted 30 of the most dominant themes from the published literature. Fig. 3 shows how the top five topics have changed over the past 78 years. Among them, topic 12 (main keywords included "treatment,” "patients,” and “infection”) was the most popular, followed by topic 24 (main keywords included “treatment,” “patients,” and “infection”), topic 2 (main keywords included “health,” “risk,” and “smoking,”), and topic 25 (main keywords included “metastatic” and "lesions”). Due to the new crown epidemic, the number of published studies on topic 22 (main keywords included “covid,” “pandemic,” and “studies") has significantly increased since 2019.

**Figure 4:**
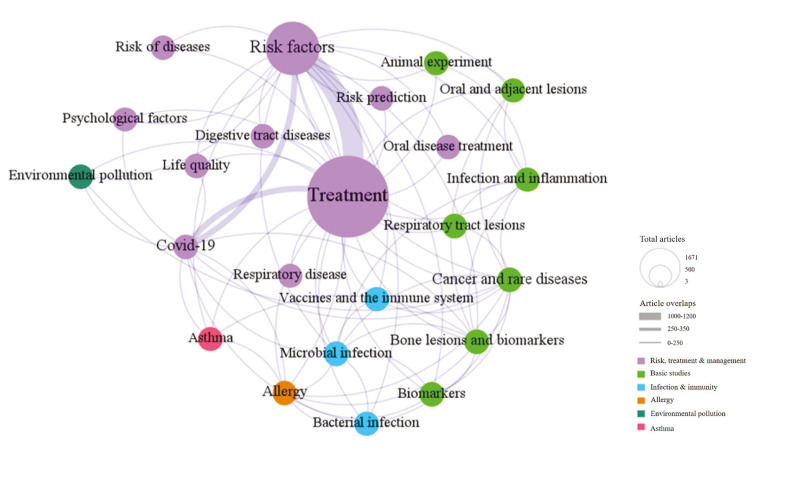
Network diagram of clustering of LDA study topics, showing the “Risk, treatment and management” (purple), “Basic studies” (light green), “Infection and immunity” (blue) “Allergy” .(orange), “Environmental pollution” (dark green), and “Asthma” (pink) clusters. LDA, latent Dirichl*et al*location [1-100].

Network analysis can be used to cluster research topics with high similarity as well as those that are interrelated to explore the strength of connections between them. In this study, six thematic network clusters were identified using the Louvain algorithm (Fig. 4). The literature on each network cluster showed strong interconnections.

The results of the Louvain algorithm showed that research on the association between oral periodontal diseases and COPD can be divided into six areas as follows: 1) risk, treatment, and management; 2) basic research; 3) infection and immunity; 4) allergic reactions and biological mechanisms; 5) environmental pollution; and 6) asthma. Among them, only a few studies were on allergic reactions and biological mechanisms, environmental pollution, and asthma (one study for each topic). For each theme, the size of the bubble indicates the amount of related literature, and the line between the bubbles indicates the co-occurrence of the two themes in different studies.

Within the disease risk, treatment, and management clusters, the most popular research themes were treatments and risk factors, followed by disease risk prediction, disease risk, and association studies with other oral diseases, gastrointestinal diseases, and neo-coronary pneumonia. In addition, “psychological factors” and “life treatments” were topics of interest for researchers. “Treatments” showed a strong association with “risk factors,” followed by new crown epidemic-related studies showing strong associations with “treatments” and “risk factors.”

In the basic research cluster, animal experiments; infection and inflammation; biomarkers; oral, respiratory, and skeletal lesions; tumor recurrence; and rare disease-related studies were similarly prevalent and strongly associated with each other.

Previous studies within the “infection and immunity” cluster focused on three themes: vaccines and the immune system, microbial infections, and bacterial infections. In addition, few studies focused on environmental pollution, asthma-related diseases, and allergic reactions and biological mechanisms.

## Discussion

COPD is the third leading cause of death globally and poses a huge economic burden (15). COPD often coexists with various diseases (comorbidities) that play important roles in its progression and prognosis. Understanding the relationship between COPD and its comorbidities is important for disease prevention and management. The present study provides groundbreaking insights into the risk factors, clinical studies, and mechanisms that enrich the understanding of the relationship between periodontitis and COPD.

We analyzed all the publications in PubMed and found that the number of studies related to periodontitis and COPD has been continuously increasing since the 1990s, with a surge beginning in 2020, indicating that the relationship between the two diseases continues to receive widespread attention. Two of the 10 most frequently cited publications are by Frank A. Scannapieco. This demonstrates the author's outstanding contributions to this field of research. His studies are widely cited by researchers. This finding is also consistent with the author's analysis of the three clinical research articles on periodontitis and COPD in our research group. Frank A. Scannapieco is a periodontist and microbiologist at the University at Buffalo School of Dental Medicine. He has been working on dental plaques and the connection between oral and overall health, particularly in at-risk populations. Two of the 10 most frequently cited publications are by him, demonstrating his centrality to the field. Prof. Wang Zuomin has 3 publications, which is consistent with having the most publications among the core of highly productive authors. We have been working on the relationship between periodontitis and COPD since around 2009. We have made substantial contributions to the field of periodontitis and systemic diseases. In addition, the exploration of periodontitis and COPD reflects the integration of different disciplines. As a result, highly prolific and cited publications in the field are not limited to periodontics-related journals, but also include respiratory disease journals. These journals have contributed significantly to the integration and development of different disciplines.

The results of the analysis by country show that the number of publications globally is not balanced between countries, with only two countries publishing more than 300 documents. Although China leads in publications, the number of citations is lower than other countries. It may be because most China publications have appeared only in recent years. Overall, the United States, England, and China have a relatively central role in the global cooperation network. Therefore, countries should eliminate academic barriers and strengthen mutual cooperation and exchange. Cross-country and cross-team cooperation is the key to accelerate global disciplines development.

Consistent with our findings, both diseases are predominantly prevalent in adults, middle-aged adults, and older adults (16), imposing a heavy burden on healthcare and the economy. Our findings suggest that the theme “risk factors” was a hotspot for periodontitis- and COPD-related studies. With respect to the trends in MeSH terminology, ranking of LDA topics, and thematic network analyses, risk factor-related research has increased, especially in recent years. Periodontitis and COPD are associated with multiple risk factors. Smoking is one of the most important risk factors for COPD and periodontitis. Severe periodontitis leads to an increase in the levels of C-reactive protein, interleukin- 1 (IL- 1), and IL-6, which may work synergistically with the toxins in cigarette smoke to exacerbate inflammatory changes in tissues and organs. A previous study showed that smokers with extracted teeth were almost seven times more likely to report COPD than those without tooth loss (17).

Studies within the “infection and immunity” cluster focused on microbial and bacterial infections. These are equally important risk factors for periodontitis and COPD. Periodontitis, which is closely associated with oral microorganisms, is mainly caused by plaque biofilms. Dysregulated oral microbial environments may lead to the development of chronic inflammatory diseases. Microbial migration from the oral cavity appears to occur via microinhalation and inhalation into the lung microbiome, suggesting a strong association between the microbiota in the oral and respiratory tissues of healthy individuals (18). Thus, oral microbiota is closely related to the *in vivo* homeostasis of the oral cavity and lungs. Similarly, microbial infections are risk factors for COPD, with dysbiosis of the lung microbiota and abnormal inflammatory responses being the two main causes of acute exacerbation of COPD (19). Lin *et al*. used 16s rRNA gene sequencing to compare the microbiota of subgingival plaques and gingival sulcus samples from healthy controls, patients with periodontitis, patients with COPD, and patients with periodontitis and COPD. The subgingival plaque reflected differences in the subgingival microbiota of patients with COPD compared to gingival sulcus fluid (13). Similar to the study by Lin *et al*., Wu *et al*. analyzed subgingival plaques obtained from 105 patients, and their results suggested that an increase in periodontitis-associated microbiota may be associated with COPD (20). Although microbial infections play an important role in the association between periodontitis and COPD, a direct causal relationship between the two diseases has not been confirmed. However, microbial-based therapy is a promising future approach that can be applied in colony transplantation therapy. This requires theoretical support for the development of new methods for predicting patient responses to different colony-based treatments.

When ranking the number of occurrences of all MeSH terms, we found that “Pandemics” ranked third and remained among the top 15 most popular MeSH terms over the last 8 years. Meanwhile, “Cross-Sectional Studies,” “Retrospective Studies,” and “Surveys and Questionnaires” were among the top 10 epidemiology- related MeSH terms from 2016 to 2023, while “Case-Control Studies” and “Follow-Up Studies” were also among the top 10 terms from 2006 to 2015. We found studies that associated periodontitis to lung disease since the 1940s, but they were limited to tuberculosis and pulmonary embolism. By 1979, several genetic polymorphisms (Pi types) in alpha 1-antitrypsin were detected, some of which were found to be associated with susceptibility to emphysema (a subtype of COPD) as well as chronic periodontitis (21). It was not until 1994 that Travis *et al*. provided more details on the possible association between periodontitis and COPD (emphysema subtype) (22). Since then, a growing body of epidemiological data has confirmed a correlation between these two chronic diseases. Recently, a large prospective UK Biobank cohort study found a clear association between periodontal disease and airflow limitation, suggesting a strong correlation between periodontal disease and the risk of COPD (23). A population-based retrospective cohort study by Shen *et al*. showed that, compared to the general population, patients with COPD were at a higher risk of developing periodontal disease. The ratio of the risk of periodontal disease to COPD control was also confirmed. Additionally, patients treated with corticosteroids were at a higher risk of developing periodontal disease (24). Kenji *et al*., Chen *et al*., and Lee *et al*. suggested that the deterioration of periodontal status or moderate-to-severe periodontitis may be important risk factors for the rapid decline in lung function in adults (25-27). In addition, Liu *et al*. showed that improving periodontal health and oral hygiene might be a potential method against COPD exacerbation (14). Therefore, the promotion and maintenance of optimal periodontal health and timely treatment may effectively mitigate the impairment of lung function that leads to COPD, which is also relevant to “Oral Health” in MeSH.

The topic “environmental pollution” included only a few studies when based on the six broadly delineated areas of research on the association between periodontal disease and COPD or on the “infection and immunity” cluster. A large body of evidence shows that environmental pollutants adversely affect health, particularly by increasing systemic inflammation and oxidative stress (28). Air pollutants can enter the body through the oral cavity, where they are inhaled into the lungs or come into direct contact with periodontal tissues and are then ingested with food or water. Therefore, air pollution is a potential risk factor for periodontitis. Yang *et al*. also found that periodontitis-affected patients were more susceptible to particulate matter (PM) exposure-induced systemic effects than healthy individuals (29). Similarly, higher concentrations of PM2.5, PM10, and NO2 were significantly associated with the prevalence of COPD, leading to lower lung function (30). Therefore, the effects of environmental pollution on periodontitis and COPD require further investigation.

The top two MeSH terms in the last eight years were “COVID- 19” and “SARS-CoV-2,” which is in line with the broader context of infectious disease epidemics. The 2019 coronavirus disease (COVID- 19), caused by severe acute respiratory syndrome coronavirus 2 (SARS-CoV-2), was declared a pandemic by the World Health Organization. Similar to the relationship between COPD and periodontal disease, oral infections caused by periodontitis may affect the respiratory system through the oral-pulmonary pathway and increase the risk of COVID- 19 infection and/or symptoms (31). This suggests that oral health management is an effective measure to prevent these diseases.

The determination of the mechanism by which periodontitis affects COPD requires the use of animal models. Accordingly, “Animals” was among the top 6 MeSH terms in our study. Inoculating the trachea of mice with periodontal bacteria (*Fusobacterium nucleatum*) stimulated the production of pro-inflammatory cytokines in human bronchial and lung epithelial cells as well as in serum, which also occurred during elastase-induced exacerbation of COPD in emphysema-induced mice (32). Tian *et al*. found that long-term ligation combined with a mouse model coated with *Porphyromonas *gingivalis** as well as cytokines induced lung inflammation (33).

This study has some limitations. We acknowledge that the use of a single database, specifically PubMed/MEDLINE, presents a methodological constraint. This choice may have excluded relevant studies indexed in other databases such as Embase, potentially limiting the comprehensiveness of our analysis. Although PubMed hosts the highest quality peer-reviewed research, excluding irrelevant non-peer-reviewed publications, a more comprehensive and detailed understanding of the literature can be achieved by exploring additional databases such as Embase. However, we chose PubMed/MEDLINE due to its extensive coverage of biomedical literature and its robust indexing of peer-reviewed articles. Additionally, PubMed/MEDLINE is widely recognized for its accessibility and reliability, making it a valuable resource for conducting bibliometric analyses. Moreover, the study included only English-language papers and is therefore subjected to source bias. Studies published after August 2023 were not included in the statistics, which may have affected the trends observed in the study.

## Conclusions

In conclusion, this bibliometric analysis provides a thorough examination of the relationship between periodontitis and COPD. The increasing research output underscores the growing global interest in understanding the interplay between these chronic conditions. Our findings underscore the imperative for continued investigation into the causal pathways linking periodontitis and COPD. Shared risk factors, notably smoking and microbial infections, warrant further exploration as potential targets for preventive interventions and therapeutic strategies. Moreover, the emerging role of environmental pollution in exacerbating both conditions necessitates focused research efforts to elucidate its mechanisms and mitigate its impact on disease progression. As research in this field evolves, interdisciplinary collaboration and standardized methodologies will be essential for advancing our understanding of periodontitis-COPD associations and developing evidence-based guidelines for clinical practice.

## Figures and Tables

**Table 1:** Top 100 cited authors in periodontitis and COPD.

Rank	Author	Documents	Citations	Average Citation/ Publication
1	Wang ZM	10	386	38.6
2	Song YQ	8	379	47.38
3	Zhang J	10	378	37.8
4	Zhou X	8	365	45.63
5	Kao CH	10	116	11.6
6	Reis, Catia	1	268	268.00
7	Zhang, Jing	5	263	52.60
8	Liu, Zhiqiang	2	165	82.50
9	Zhang, Liangqiong	2	147	73.50
10	Han, Jing	3	135	45.00
11	Herzberg, Mark C.	1	129	129.00
12	Linden, Gerry J.	1	129	129.00
13	Wang, Chen	2	109	54.50
14	Carvalho-Filho, Paulo Cirino	1	104	104.00
15	Da Cruz, Simone Seixas	1	104	104.00
16	Gomes-Filho, Isaac Suzart	1	104	104.00
17	Hintz, Alexandre Marcelo	1	104	104.00
18	Lyrio, Amanda Oliveira	1	104	104.00
19	Morais Godoy Figueiredo, Ana Claudia	1	104	104.00
20	Passos-Soares, Johelle de Santana	1	104	104.00
21	Pereira, Mauricio Gomes	1	104	104.00
22	Scannapieco, Frank	1	104	104.00
23	Trindade, Soraya Castro	1	104	104.00
24	Sun, Zheng	1	93	93.00
25	Mammen, Manoj J.	1	90	90.00
26	Scannapieco, Frank A.	1	90	90.00
27	Sethi, Sanjay	1	90	90.00
28	Baser, Ulku	2	78	39.00
29	Kiyan, Esen	2	78	39.00
30	Kucukcoskun, Meric	2	78	39.00
31	Oztekin, Gorkem	2	78	39.00
32	Yalcin, Funda	2	78	39.00
33	Hu, Frank B.	1	75	75.00
34	Zhang, Wei	1	72	72.00
35	Fan, Hong	1	58	58.00
36	Si, Yan	1	58	58.00
37	Bjortuft, O.	1	55	55.00
38	Geiran, O.	1	55	55.00
39	Leuckfeld, I.	1	55	55.00
40	Lund, M. B.	1	55	55.00
41	Obregon-Whittle, M. V.	1	55	55.00
42	Olsen, I.	1	55	55.00
43	Fukuyama, S.	1	54	54.00
44	Furuta, M.	1	54	54.00
45	Hata, J.	1	54	54.00
46	Inoue, H.	1	54	54.00
47	Matsumoto, K.	1	54	54.00
48	Nakanishi, Y.	1	54	54.00
49	Ninomiya, T.	1	54	54.00
50	Ogata, H.	1	54	54.00
51	Shibata, Y.	1	54	54.00
52	Shimazaki, Y.	1	54	54.00
53	Suma, S.	1	54	54.00
54	Takeshita, T.	1	54	54.00
55	Takeuchi, K.	1	54	54.00
56	Yamashita, Y.	1	54	54.00
57	Lin, H. -C.	1	48	48.00
58	Sheu, J. -J.	1	48	48.00
59	Stockley, Robert A.	1	45	45.00
60	Usher, Adam K. H.	1	45	45.00
61	Chung, Jae Ho	1	43	43.00
62	Hwang, Hee-Jin	1	43	43.00
63	Kim, Sun-Hyun	1	43	43.00
64	Kim, Tae Ho	1	43	43.00
65	Bardapurkar, Suhas Jagannath	1	42	42.00
66	Borkar, Mangala Sonawani	1	42	42.00
67	Doiphode, Satish Shripad	1	42	42.00
68	Mute, Bhumika Ramchandra	1	42	42.00
69	Peter, Kalpak Prafulla	1	42	42.00
70	Raje, Dhananjay Vasant	1	42	42.00
71	Bretz, W	1	40	40.00
72	Chen, Yi-Hua	1	40	40.00
73	Corby, P	1	40	40.00
74	Crapo, Ro	1	40	40.00
75	Jensen, R	1	40	40.00
76	Katancik, Ja	1	40	40.00
77	Keller, Joseph J.	1	40	40.00
78	Kritchevsky, S	1	40	40.00
79	Lin, Herng-Ching	1	40	40.00
80	Lin, Mei	3	40	13.33
81	Newman, Ab	1	40	40.00
82	Rubin, Sm	1	40	40.00
83	Waterer, G	1	40	40.00
84	Weyant, Rj	1	40	40.00
85	Wu, Chuan-Song	1	40	40.00
86	Chen, Jiazhen	1	35	35.00
87	Chen, Yulin	1	35	35.00
88	Cui, Chenghao	1	35	35.00
89	Wang, Xuyang	1	35	35.00
90	Winning, Lewis	2	35	17.50
91	Wu, Jing	1	35	35.00
92	Wu, Xingwen	1	35	35.00
93	Xu, Meng	1	35	35.00
94	Yu, Liying	1	35	35.00
95	Zhang, Wenhong	1	35	35.00
96	Zhu, Danting	1	35	35.00
97	Cheng, Cheng	2	33	16.50
98	Wang, Songlin	2	33	16.50
99	Wang, Jitian	2	31	15.50
100	Chapple, Iain	1	28	28.00

**Table 2:** Top 100 cited references of publications in periodontitis and COPD.

Title	Type	First author	Source	year	Frequency
The immune response to Prevotella bacteria in chronic inflammatory disease.	Article	Jeppe Madura Larsen	Immunology	2017	537
Systematic review of the association between respiratory diseases and oral health	Review	Amir Azarpazhooh	Journal of Periodontology	2006	375
Role of oral bacteria in respiratory infection	Review	Frank A. Scannapieco	Journal of Periodontology	1999	354
Associations between periodontal disease and risk for nosocomial bacterial pneumonia and chronic obstructive pulmonary disease. A systematic review	Review	Frank A Scannapieco	Journal of Periodontology	2003	214
Potential associations between chronic respiratory disease and periodontal disease: analysis of National Health and Nutrition Examination Survey III	Article (secondary research)	Frank A. Scannapieco	Journal of Periodontology	2001	173
Periodontitis is an inflammatory disease of oxidative stress: We should treat it that way	Review	Fábio Sá Carneiro Sczepanik	Periodontology 2000	2020	136
Periodontitis-systemic disease associations in the presence of smoking--causal or coincidental?	Review	P P Hujoel	Periodontology 2000	2000	109
Periodontitis and respiratory diseases: A systematic review with meta-analysis	Meta-Analysis (secondary research)	Isaac Suzart Gomes Filho	Oral Diseases	2020	96
Effects of periodontal treatment on lung function and exacerbation frequency in patients with chronic obstructive pulmonary disease and chronic periodontitis: A 2-year pilot randomized controlled trial	Article (primary research)	Zhou Xuan	Journal of Clinical Periodontology	2014	92
The association between alveolar bone loss and pulmonary function: the VA Dental Longitudinal Study	Article	C Hayes	Journal of Periodontology	1998	91
Periodontal Pathogens as Risk Factors of Cardiovascular Diseases, Diabetes, Rheumatoid Arthritis, Cancer, and Chronic Obstructive Pulmonary Disease-Is There Cause for Consideration?	Review	Denis Bourgeois	Microorganisms	2019	85
Periodontal disease and risk of chronic obstructive pulmonary disease: a meta-analysis of observational studies	Meta-Analysis	Zeng Xian-Tao	PLoS One	2012	76
Effects of periodontal treatment on lung function and exacerbation frequency in patients with chronic obstructive pulmonary disease and chronic periodontitis: a 2-year pilot randomized controlled trial	Randomized Controlled Trial	Zhou xuan	Journal of Clinical Periodontology	2014	75
Periodontitis and respiratory diseases: A systematic review with meta-analysis	Meta-Analysis	Isaac Suzart Gomes-Filho	Oral Diseases	2020	75
Periodontal health, oral health behaviours, and chronic obstructive pulmonary disease	Article (primary research)	Wang Zuomin	Journal of Clinical Periodontology	2009	73
Oral hygiene, periodontal health and chronic obstructive pulmonary disease exacerbations	Article (primary research)	Liu Zhiqiang	Journal of Clinical Periodontology	2012	70
Is periodontitis a comorbidity of COPD or can associations be explained by shared risk factors/behaviors?	Review	Stephanie Hobbins	International Journal of Chronic Obstructive Pulmonary Disease	2017	64
Periodontal health and systemic disorders	Review	Yen-Tung A Teng	Journal Of The Canadian Dental Association	2002	59
Initial periodontal treatment for prevention of chronic obstructive pulmonary disease exacerbations	Article (primary research)	Meric Kucukcoskun	Journal of Clinical Periodontology	2013	58
Periodontal health, oral health behaviours, and chronic obstructive pulmonary disease	Article	Wang Zuomin	Journal Of Clinical Periodontology	2009	57
Periodontitis and systemic diseases: a record of discussions of working group 4 of the Joint EFP/AAP Workshop on Periodontitis and Systemic Diseases	Article	Gerry J Linden	Journal of Clinical Periodontology	2013	57
Oral hygiene, periodontal health and chronic obstructive pulmonary disease exacerbations	Comparative Study	Liu Zhiqiang	Journal Of Clinical Periodontology	2012	53
Potential role of periodontal infection in respiratory diseases - a review	Review	M Bansal	Journal of Medicine and Life	2013	52
Epidemiologic associations between periodontal disease and chronic obstructive pulmonary disease	Meta-Analysis	R I Garcia	Journal of Periodontology	2001	49
Initial periodontal treatment for prevention of chronic obstructive pulmonary disease exacerbations	Clinical Trial	Meric Kucukcoskun	Journal Of Periodontology	2013	44
Severe chronic obstructive pulmonary disease: association with marginal bone loss in periodontitis	Article	I Leuckfeld	The Lancet Respiratory Medicine	2008	43
Investigating the Association Between Periodontal Disease and Risk of Pancreatic Cancer	Article	Jeffrey S Chang	Pancreas	2016	43
Is periodontitis a comorbidity of COPD or can associations be explained by shared risk factors/behaviors?	Review	Stephanie Hobbins	International Journal of Chronic Obstructive Pulmonary Disease	2017	41
Cigarette smoking, periodontal disease: and chronic obstructive pulmonary disease	Article	Jeffrey J Hyman	Journal Of Periodontology	2004	40
Association between periodontitis and chronic obstructive pulmonary disease in a Chinese population	Article	Si Yan	Journal Of Periodontology	2012	37
Periodontal inflammation: from gingivitis to systemic disease?	Article	Frank A Scannapieco	Compend Contin Educ Dent	2004	37
Associations Between Periodontitis and Chronic Obstructive Pulmonary Disease: The 2010 to 2012 Korean National Health and Nutrition Examination Survey	Article	Jae Ho Chung	Journal Of Periodontology	2016	36
A cohort study of the impact of tooth loss and periodontal disease on respiratory events among COPD subjects: modulatory role of systemic biomarkers of inflammation	Clinical Trial	Silvana P Barros	PLoS One	2013	36
Association between obstructive sleep apnoea and chronic periodontitis: a population-based study	Article	Joseph J Keller	Journal of Clinical Periodontology	2013	35
Periodontitis and systemic diseases: a record of discussions of working group 4 of the Joint EFP/AAP Workshop on Periodontitis and Systemic Diseases	Article	Gerry J Linden	Journal Of Periodontology	2013	35
16S rDNA-based metagenomic analysis of dental plaque and lung bacteria in patients with severe acute exacerbations of chronic obstructive pulmonary disease	Observational Study	L Tan	Journal Of Periodontal Research	2014	35
Periodontitis Is Associated with Chronic Obstructive Pulmonary Disease.	Article	K Takeuchi	Journal of Dental Research	2019	33
The link between periodontal disease and cardiovascular disease: How far we have come in last two decades ?	Article	Prasad Dhadse	Journal of Indian Society of Periodontology	2010	33
Association between multiple sclerosis and chronic periodontitis: a population-based pilot study	Clinical Trial	J-J Sheu	European Journal Of Neurology	2013	32
Association between periodontal disease and chronic obstructive pulmonary disease: a reality or just a dogma?	Clinical Trial	Kalpak Prafulla Peter	Observational Study	2013	31
The role of proteolytic enzymes in the development of pulmonary emphysema and periodontal disease	Review	J Travis	American Journal of Respiratory and Critical Care Medicine	1994	30
Association between respiratory disease in hospitalized patients and periodontal disease: a cross-sectional study	Clinical Trial	Nikhil Sharma	Journal Of Periodontology	2011	29
Role of periodontal therapy in management of common complex systemic diseases and conditions: An update	Review	Amarpreet Sabharwal	Periodontol 2000	2018	29
Risk of Periodontal Diseases in Patients With Chronic Obstructive Pulmonary Disease: A Nationwide Population-based Cohort Study	Observational Study	Shen Te-Chun	Medicine (Baltimore)	2015	28
Compartment differences of inflammatory activity in chronic obstructive pulmonary disease	Article	Ji jie	RESPIRATORY RESEARCH	2014	28
16S rDNA analysis of periodontal plaque in chronic obstructive pulmonary disease and periodontitis patients	Article	Wu Xingwen	Journal of Oral Microbiology	2017	26
Pathological Characteristics of Periodontal Disease in Patients with Chronic Kidney Disease and Kidney Transplantation	Review	Mineaki Kitamura	International Journal of Molecular Sciences	2019	26
Periodontitis as a potential risk factor for chronic obstructive pulmonary disease: a retrospective study	Comparative Study	Vikas Deo	Indian Journal of Dental Research (IJDR)	2009	25
Association between periodontal diseases and cardiovascular diseases, diabetes and respiratory diseases: Consensus report of the Joint Workshop by the European Federation of Periodontology (EFP) and the European arm of the World Organization of Family Doctors (WONCA Europe)	Review	David Herrera	Journal of Clinical Periodontology	2023	25
Patients with Chronic Obstructive Pulmonary Disease Suffer from Worse Periodontal Health-Evidence from a Meta-Analysis	Meta-Analysis	Shi Quan	Frontiers in Physiology	2018	24
The clinical and inflammatory relationships between periodontitis and chronic obstructive pulmonary disease.	Article	Elizabeth Sapey	Journal Of Clinical Periodontology	2020	24
Causal relationship between periodontitis and chronic obstructive pulmonary disease	Article	Surya J Prasanna	Journal of Indian Society of Periodontology	2011	24
The link between chronic periodontitis and COPD: a common role for the neutrophil?	Review	Adam K H Usher	BMC Medicine	2013	24
From the Acta Prize Lecture 2014: the periodontal-systemic connection seen from a microbiological standpoint	Review	Ingar Olsen	ACTA ODONTOLOGICA SCANDINAVICA	2015	24
The relationship between periodontal diseases and respiratory diseases	Article	Frank A Scannapieco	DENT J-BASEL/DENTISTRY JOURNAL	2003	24
Relationship among clinical periodontal, microbiologic parameters and lung function in participants with chronic obstructive pulmonary disease	Article	Tan Lisi	Journal Of Periodontology	2019	22
Periodontal health and quality of life in patients with chronic obstructive pulmonary disease	Multicenter Study	Zhou xuan	The Lancet Respiratory Medicine	2011	22
Periodontal health and serum, saliva matrix metalloproteinases in patients with mild chronic obstructive pulmonary disease	Article	E Yıldırım	Journal Of Periodontal Research	2013	20
Periodontitis and airway obstruction	Article	James A Katancik	Journal Of Periodontology	2005	20
Relationship between periodontitis-related antibody and frequent exacerbations in chronic obstructive pulmonary disease	Article	Tamaki Takahashi	PLoS One	2012	19
Periodontal disease and targeted prevention using aMMP-8 point-of-care oral fluid analytics in the COVID-19 era	Article	Ismo T Räisänen	MEDICAL HYPOTHESES	2020	19
Periodontal Treatment Reduces Risk of Adverse Respiratory Events in Patients With Chronic Obstructive Pulmonary Disease: A Propensity-Matched Cohort Study	Clinical Trial	Shen Te-Chun	Medicine (Baltimore)	2016	19
The Associations between Periodontitis and Respiratory Disease	Review	S A Moghadam	Journal of Nepal Health Research Council	2017	18
Serum levels of 25-hydroxyvitamin D, oral health and chronic obstructive pulmonary disease	Article	Zhou xuan	Journal Of Clinical Periodontology	2012	18
Chronic periodontitis and reduced respiratory function	Article	Lewis Winning	Journal Of Clinical Periodontology	2019	16
Is COPD associated with periodontal disease? A population-based study in Spain	Article	Ana Lopez-de-Andrés	International Journal of Chronic Obstructive Pulmonary Disease	2018	15
The relationship of alpha1-antitrypsin to inflammatory periodontal disease	Comparative Study	R J Peterson	Journal Of Periodontology	1979	15
Periodontal status and oral health behavior in hospitalized patients with chronic obstructive pulmonary disease	Article	Neeta Vijay Bhavsar	Journal of Natural Science, Biology and Medicine	2015	15
Periodontal disease increases risk for chronic obstructive pulmonary disease	Article	Karla Ledić	Coll Antropol	2013	15
The association between periodontal disease and chronic obstructive pulmonary disease: a case control study	Clinical Trial	Görkem Öztekin	Copd-Journal of Chronic Obstructive Pulmonary Disease	2014	14
Periodontal Status and Microbiologic Pathogens in Patients with Chronic Obstructive Pulmonary Disease and Periodontitis: A Case-Control Study	Article	Zhou Xuan	International Journal of Chronic Obstructive Pulmonary Disease	2020	13
The association between respiratory diseases and periodontitis: A systematic review and meta-analysis.	Review	Ana Molina	Journal of Clinical Periodontology	2023	13
Periodontal pathogens and respiratory diseases- evaluating their potential association: a clinical and microbiological study	Clinical Trial	S Vadiraj	The journal of contemporary dental practice	2013	13
The association between dental health and nutritional status in chronic obstructive pulmonary disease	Article	Takeshi Terashima	Chronic Respiratory Disease	2017	12
Periodontal status and chronic obstructive pulmonary disease (COPD) exacerbations: a systematic review	Article	Niamh Kelly	BMC Oral Health	2021	12
Vitamin D reduces the serum levels of inflammatory cytokines in rat models of periodontitis and chronic obstructive pulmonary disease	Article	Han jing	International Journal of Oral Science	2019	12
Periodontitis and Airway Obstruction	Article	James A Katancik	Journal Of Periodontology	2005	12
Periodontal disease increases the risk for onset of systemic comorbidities in dental hospital attendees: An 18-year retrospective cohort study	Clinical Trial	Zhao Dan	Journal Of Periodontology	2019	12
Periodontitis increases the risk of respiratory disease mortality in older patients	Article	Qian Yifeng	Experimental Gerontology	2020	12
Saliva Microbiome Changes in Patients With Periodontitis With and Without Chronic Obstructive Pulmonary Disease	Article	Lin Mei	Frontiers in Cellular and Infection Microbiology	2020	10
Periodontal sources of citrullinated antigens and TLR agonists related to RA	Review	Ljubomir Vitkov	Autoimmunity	2018	10
Effect of periodontal therapy on COPD outcomes: a systematic review	Review	Ioulianos Apessos	BMC Pulmonary Medicine	2021	10
Pulmonary disease and periodontal health: a meta-analysis.	Meta-Analysis	Wu ZeSheng	Sleep And Breathing	2022	9
Individuals with chronic obstructive pulmonary disease (COPD) may be more likely to have more severe periodontal disease than individuals without COPD	Comment	Frank A Scannapieco	Journal of Evidence-Based Dental Practice	2014	9
Association between indices of clinically-defined periodontitis and self-reported history of systemic medical conditions	Article	Nikolaos A Chrysanthakopoulos	J Investig Clin Dent	2016	9
Periodontitis May Be Associated With Respiratory Diseases Such as Asthma, COPD, and Pneumonia	Comment	Niamh Kelly	Journal Of Evidence-based Dental Practice	2020	8
Advanced Dental Cleaning is Associated with Reduced Risk of COPD Exacerbations - A Randomized Controlled Trial	Randomized Controlled Trial	Josefin Sundh	International Journal of Chronic Obstructive Pulmonary Disease	2021	8
Fusobacterium nucleatum exacerbates chronic obstructive pulmonary disease in elastase-induced emphysematous mice	Article	Ryuta Suzuki	FEBS Open Bio	2022	8
Patients with chronic obstructive pulmonary disease: management considerations for the dental team	Clinical Trial	J Devlin	BRITISH DENTAL JOURNAL	2014	8
Associations between Periodontitis and COPD: An Artificial Intelligence-Based Analysis of NHANES III	Clinical Trial	Andreas Vollmer	Journal Of Clinical Medicine	2022	8
Effects of periodontal instrumentation on quality of life and illness in patients with chronic obstructive pulmonary disease: a pilot study	Randomized Controlled Trial	Brooke E Agado	International Journal Of Dental Hygiene	2012	8
The role of Th17 cells: explanation of relationship between periodontitis and COPD?	Review	Liu Jiaohong	Inflammation Research	2022	8
The impact of periodontitis in the course of chronic obstructive pulmonary disease: Pulmonary and systemic effects	Article	Ellen Perim Rosa	LIFE SCIENCES	2020	7
Periodontitis modifies the association between smoking and chronic obstructive pulmonary disease in Japanese men	Article	Jane Harland	International Journal of Oral Science	2018	7
Periodontal Diseases: Major Exacerbators of Pulmonary Diseases?	Review	Bakey Kouanda	Bmc Pulmonary Medicine	2021	7
Impact of Non-surgical Periodontal Therapy on Pulmonary functions, Periodontal Health and Salivary Matrix Metalloproteinase-8 of COPD Patients with Chronic Periodontitis: A Clinico-biochemical Study	Clinical Trial	Sakshi Sharma	Turkish Thoracic Journal	2021	7
The association between periodontitis and lung function: Results from the National Health and Nutrition Examination Survey 2009 to 2012	Article	Chen Hongru	Journal Of Periodontology	2022	7
Oral Candidal Load and Oral Health Status in Chronic Obstructive Pulmonary Disease (COPD) Patients: A Case-Cohort Study	report	Shahnawaz Khijmatgar	Biomed Research International	2021	6
Prevalence of periodontitis in patients with pulmonary disease: A cross-sectional survey in the industrial district of India	Article	Tanushree Rastogi	Journal of Indian Society of Periodontology	2019	6
Association of periodontal status with lung function in patients with and without chronic obstructive pulmonary disease visiting a medical hospital in Pune: A comparative study	Clinical Trial	Nikhil Bomble	Journal of Indian Society of Periodontology	2020	3

**Table 3:** Ranking of MeSH word occurrences (1945-2023).

Rank	MeSH	Record of occurrence in publications
1	COVID-19	455
2	SARS-CoV-2	311
3	Pandemics	210
4	Risk Factors	193
5	Animals	190
6	Diagnosis, Differential	187
7	Lung Neoplasms	173
8	Cross-Sectional Studies	154
9	Gingivitis	133
10	Histiocytosis, Langerhans-Cell	126
11	Pulmonary Disease, Chronic Obstructive	125
12	Asthma	112
13	Oral Health	109
14	Gingival Diseases	108
15	Gingival Neoplasms	107
16	Retrospective Studies	99
17	Lung	92
18	Smoking	92
19	Eosinophilic Granuloma	89
20	Case-Control Studies	85

**Table 4:** Ranking of the number of occurrences of MeSH words every 10 years.

Rank	1945-1955	1956-1965	1966-1975	1976-1985	1986-1995	1996-2005	2006-2015	2016-2023
1	Gingival Diseases	Pathology	Radiography	Histiocytosi, Langerhans-Cell	Diagnosis, Differential	Diagnosis, Differential	Risk Factors	COVID-19
2	Gingiva	Gingiva	Oral Manifestations	Diagnosis, Differential	Histiocytosis, Langerhans-Cell	Risk Factors	Diagnosis, Differential	SARS-CoV-2
3	Eosinophilic Granuloma	Gingival Diseases	Diagnosis, Differential	Gingivitis	Lung Neoplasms	Lung Neoplasms	Lung Neoplasm	Pandemics
4	Tuberculosis	Histiocytosis, Langerhans-Cell	Eosinophilic Granuloma	Gingival Diseases	Gingivitis	Granuloma, Giant Cell	Animals	Cross-Sectional Studies
5	Neoplasms	Granulomatosis with Polyangiitis	Granuloma, Giant Cell	Gingival Neoplasms	Eosinophilic Granuloma	Gingival Neoplasms	Gingival Neoplasm	Risk Factors
6	Gingival Neoplasms	Granuloma, Giant Cell	Gingival Diseases	Granuloma, Giant Cell	Granulomatosis with Polyangiitis	Smoking	Follow-Up Studies	Animals
7	Tuberculosis, Pulmonary	Gingivitis	Histiocytosis, Langerhans-Cell	Lung Neoplasms	Gingival Neoplasms	Chronic Disease	Pulmonary Disease, Chronic Obstructive	Oral Health
8	Pneumonia	Diagnosis, Differential	Biopsy	Asthma	Animals	Cardiovascular Diseases	Asthma	Pulmonary Disease, Chronic Obstructive
9	Jaw Diseases	Tuberculosis	Lung Neoplasms	Eosinophilic Granuloma	Granuloma, Giant Cell	Mouth Diseases	Case-Control Studies	Surveys and Questionnaires
10	Giant Cell Tumors	Pneumonia	Gingivitis	Mouth Diseases	Mouth Diseases	Histiocytosis, Langerhans-Cell	Gingivitis	Retrospective Studies
11	Hemoptysis	Tuberculosis, Pulmonary	Pneumonia	Saliva	Gingival Diseases	Eosinophilic Granuloma	Smoking	Coronavirus Infections
12	Hematemesis	Eosinophilic Granuloma	Animals	Lung Diseases	Radiography	Pregnancy	Gingival Diseases	Pneumonia, Viral
13	Hemorrhage	Maxilla	Neoplasm Metastasis	Granulomatosis with Polyangiitis	Lung Diseases	Pneumonia, Bacterial	Oral Hygiene	Lung
14	Melena	Anti-Bacterial Agents	Tuberculosis, Pulmonary	Gingiva	Lung	Radiography	Periodontal Index	Betacoronavirus
15	Gingival Hemorrhage	Radiography, Dental	Mouth Diseases	Mandibular Diseases	Pneumonia	Animals	Treatment Outcome	Asthma
